# A uniformly convergent numerical scheme for solving singularly perturbed differential equations with large spatial delay

**DOI:** 10.1007/s42452-022-05203-9

**Published:** 2022-11-08

**Authors:** Ababi Hailu Ejere, Gemechis File Duressa, Mesfin Mekuria Woldaregay, Tekle Gemechu Dinka

**Affiliations:** 1 Department of Applied Mathematics, Adama Science and Technology University, 1888 Adama, Ethiopia; 2 Department of Mathematics, Jimma University, 378 Jimma, Ethiopia

**Keywords:** Singularly perturbation, Boundary layers, Large delay, $\theta$-Method, Uniform convergence, 65M06, 65M12, 65M22, 65M25

## Abstract

In this study, a parameter-uniform numerical scheme is built and analyzed to treat a singularly perturbed parabolic differential equation involving large spatial delay. The solution of the considered problem has two strong boundary layers due to the effect of the perturbation parameter, and the large delay causes a strong interior layer. The behavior of the layers makes it difficult to solve such problem analytically. To treat the problem, we developed a numerical scheme using the weighted average ($\theta$-method) difference approximation on a uniform time mesh and the central difference method on a piece-wise uniform spatial mesh. We established the Stability and convergence analysis for the proposed scheme and obtained that the method is uniformly convergent of order two in the temporal direction and almost second order in the spatial direction. To validate the applicability of the proposed numerical scheme, two model examples are treated and confirmed with the theoretical findings.

## Introduction

A differential equation is said to be a delay differential equation (DDE) if the evolution of a dependent variable or its derivatives appears depending on the values of the previous state. DDE can manifest itself in different forms, such as constant delay, variable delay, state-dependent delay, distributed delay and so on [1]. In science and engineering, DDEs are used to model a variety of real-world phenomena, such as variation problems in control theory [2], stochastic neuronal movement depolarization model [3], mathematical modelling of HIV-1 infection [4], to model COVID-19 pandemic [5] and others.

A class of differential equations, which involves a small positive parameter $\varepsilon$ (often $0<\varepsilon \ll 1$) multiplied by the term with highest order derivative and one or more shifting parameters is said to be a singularly perturbed delay differential equation. The delay parameter may be considered as larger or smaller than the perturbation parameter. If the delay parameter exceeds the perturbation parameter, then the problem is said to be a singularly perturbed problem with large delay. On the other hand, if the delay parameter is less than the perturbation parameter, then the problem is said to be a singularly perturbed problem with small delay [6–8].

The presence of the perturbation parameter causes the problem’s solution to change rapidly in certain regions and slowly in other regions of the domain. Regions where the solution changes rapidly are known as the inner region and those where the solution changes slowly are said to be the outer regions. Perturbation problems may be classified as singular and regular. A regular perturbation problem is a problem whose solution varies smoothly as $\varepsilon$ approaches zero, whereas singular perturbation problem is the one in which the solution changes precipitously in some way as $\varepsilon$ approaches zero. Thus, in the study of singular perturbation problems, naively considering $\varepsilon$ to be zero changes the very nature of the problem. The rapid changing behavior of the layers makes it difficult to find an analytical solution. Also, classical numerical methods do not give a simplified and satisfactory result as they do not take the characteristics of the solutions in the boundary or interior layer into account, which causes a significant discrepancy between the numerical solution and the actual solution. To overcome these kinds of computational problems, there is a need of parameter independent numerical methods [9].

Numerous research works are available in literature to obtain suitable numerical techniques for singularly perturbed problems,. For instance, Kumar [10] proposed a collocation method for singularly perturbed turning point problems involving boundary/interior layers. Kumar and Kumari [11] solved singularly perturbed problems with integral boundary condition by constructing a parameter-uniform collocation scheme. Cimen and Amiraliyev [12] solved singularly perturbed problems based on a piecewise uniform mesh of Shishkin type. Cimen [13] treated singularly perturbed differential equation with delay and advance by constructing a scheme by the method of integral identities with the use of interpolating quadrature rules. Kumar and Kanth [14] solved time dependent singularly perturbed differential equation using tension spline on a non-uniform Shishkin mesh. Adilaxmi and Reddy [15] solved singularly perturbed differential-difference equations applying an initial value technique via fitted nonstandard finite difference method. Musharary and Mohapatra [16] treated singularly perturbed problems with mixed arguments by constructing a hybrid parameter-uniform numerical schemes. Shakti and Mohapatra [17] presented monotone hybrid numerical scheme for a singularly perturbed convection-diffusion problem on different types of nonuniform meshes. Bansal and Sharma [18] formulated a parameter-uniform numerical scheme using fitted mesh method to solve a singularly perturbed convection-diffusion problem with shift arguments. Woldaregay and duressa [19] treated singularly perturbed problems involving both shift and advance parameters by developing a parameter-uniform numerical scheme using nonstandard finite difference method in *x*-direction and implicit Runge–Kutta method in *t*-direction. Ejere et al. [20] solved a singularly perturbed differential equation with negative shift by proposing a fitted numerical scheme via domain decomposition. Bansal and Sharma [21] developed a uniformly convergent numerical scheme for singularly perturbed reaction-diffusion problems by using implicit Euler method in the time direction and central finite difference method in the spatial direction. Kumar and Kumari [22] treated singularly perturbed reaction–diffusion problems with large delay by developing a parameter-uniform numerical scheme using the Crank–Nicolson method in time direction and the central finite difference method in the spatial direction. Singularly perturbed differential equations involving temporal delay are well solved in various research works, some of which can be referred in [23–26] for the detail discussions.

In this research work, we are motivated to develop a uniformly convergent numerical scheme to treat a singularly perturbed differential equations involving large delay in the spatial variable. To treat the problem, we developed a numerical scheme using the $\theta$-method finite difference approximation on a uniform time discretization and using the central finite difference operator on a piece-wise uniform spatial discretization. For the developed numerical scheme, we have established the stability and convergence analysis, which show that our method is convergent, regardless of the perturbation parameter. To demonstrate the validity and applicability of the obtained method, two numerical examples are treated and confirmed with the theoretical findings.

This paper is organized as follows: In Sect. 2, we describe the continuous problem. The numerical method is briefly discussed in Sect. 3. To validate the proposed numerical scheme, we treated and discussed model examples in Sect. 4. The conclusion of the paper is given in Sect. 5. Through out this study, *C* is considered as a generic positive constant which is independent of the perturbation parameter and the mesh numbers. For a given continuous function u(x,t), we defined the maximum norm as $\Vert u(x,t)\Vert =\smash {\displaystyle \max _{(x,t)\in \bar{\Omega }}}|u(x,t)|$.

## Continuous problem

Consider a singularly perturbed problem on $\Omega = \Gamma \times \Lambda = (0,2)\times (0,T]$ and $\partial \Omega =\{(x,0)\cup (0,t)\cup (2,t): x \in \bar{\Gamma }=[0, 2], t\in \bar{\Lambda }=[0, T]\}$ for a finite time *T* as

1 $$\begin{aligned} u_{t}-\varepsilon u_{xx}+p(x)u(x,t)+q(x)u(x-1,\,t)=f(x) \end{aligned}$$

subjected to the initial condition $u(x,0)=u_{0}(x),\ \ \forall x \in \bar{\Gamma }$ and the boundary conditions $u(x,t)=\gamma (x,t), \ \ \forall (x,t) \in \Omega ^{-}, \ u(2,t)=\zeta (t), \ \ \forall (2,t) \in \Omega ^{+}$, where $\Omega ^{-}=\{(x,t): x\in [-1,0], \ t \in \bar{\Lambda }\}$, $\Omega ^{+}=\{(2,t): t \in \bar{\Lambda }\}$ and $0 < \varepsilon \ll 1$.

### Basic assumptions of the continuous problem

We assume that the functions involved in the continuous problem are all smooth enough and moreover, for arbitrary positive number $\beta$, the functions *p*(*x*) and *q*(*x*) satisfy the conditions

2 $$\begin{aligned} p(x)+q(x) \ge 2\beta >0 , \ \ q(x) < 0 ,\ \ \ \ x \in \bar{\Gamma }. \end{aligned}$$

Considering the interval boundary conditions, (1) can be written as

3 $$\begin{aligned} L_{\varepsilon }u(x,t)= {\left\{ \begin{array}{ll} u_{t}-\varepsilon u_{xx}+p(x)u(x,t)=f(x)-q(x)\gamma (x-1,\,t), \, x\in (0,1],\, t\in (0,T], \\ u_{t}-\varepsilon u_{xx}+p(x)u(x,t)+q(x)u(x-1,\,t)=f(x), \, x\in (1,2),\,t\in (0,T] \end{array}\right. } \end{aligned}$$

with $u(x,0)=u_{0}(x)$ for $x\in \bar{\Gamma }$, $u(x,t)=\gamma (x,t)$ for $(x,t)\in \Omega ^{-}$, $u(2,t)=\zeta (t)$ for $t\in \bar{\Lambda }$, $u(1^{-},\,t)=u(1^{+},\,t)$ and $u_{x}(1^{-},\, t)=u_{x}(1^{+},\, t)$ .

Letting $\varepsilon =0$, we obtain the reduced form of Eq. (3) as

$$\begin{aligned} L_{0}u(x,t)= {\left\{ \begin{array}{ll} (u_{0})_{t}+p(x)u_{0}(x,t)=f(x)-q(x)\gamma (x-1,\,t), \, x\in (0,1],\, t\in (0,T], \\ (u_{0})_{t}+p(x)u_{0}(x,t)+q(x)u_{0}(x-1,\,t)=f(x),\, x\in (1,2),\, t\in (0,T]. \end{array}\right. } \end{aligned}$$

From the reduced problem, we observe that $u_{0}(x,t)$ does not necessarily satisfy $u_{0}(0,t)=\gamma (0,t)$ and $u_{0}(2,t)=\zeta (t)$. This shows that the solution involves boundary layers at the end of the domain. At $x=1$, since $u_{0}(1^{-}, t)=\frac{f(1, t)-q(1)\gamma (0^{-}, t)-(u_{0})_{t}(1, t)}{p(1)}$ and $u_{0}(1^{+}, t)=\frac{f(1, t)-q(1)u_{0}(0^{+},t)-(u_{0})_{t}(1, t)}{p(1)}$, it is not necessary that $u_{0}(1^{-},t)$ is equal to $u_{0}(1^{+},t)$. Hence, at $x=1$ the solution involves strong interior layer. For more information we may refer [22, 27]. The compatibility requirement is also imposed to be satisfied by the problem under consideration, which means that at the points $(-1,0), (0,0), (1,0)$ and (2, 0) we have

$$\begin{aligned}&u_{0}(0,0)= \gamma (0,0) , \ \ u_{0}(2,0)=\zeta (0),\\&\gamma _{t}(0,0)-\varepsilon (u_{0})_{xx}(0,0)+p(0)u_{0}(0,0)+q(0)\gamma (-1,0)=f(0),\\&\zeta _{t}(0)-\varepsilon (u_{0})_{xx}(2,0)+p(2)u_{0}(2,0)+q(2)u_{0}(1,0)=f(2). \end{aligned}$$

As a result, if the aforementioned conditions are met, the existence and uniqueness of the solution of the continuous problem on a given domain can be determined. Thus, as described in [28], for $(x,t) \in \bar{\Omega }$, we have $|u(x,t)-u_{0}(x)|\le Ct$.

### Properties of the solution and its derivatives

In this subsection, we describe the stability and bound of the analytical solution and its derivatives for the continuous problem, which are important in the analysis of the discrete problem [29].

#### Lemma 1

Let $\psi$ be a given sufficiently smooth function on $\bar{\Omega }$. If $\psi (x,t) \ge 0$, $(x,t)\in \partial \Omega$ and $L_{\varepsilon }\psi (x,t)\ge 0$, $(x, t) \in \Omega$, then we have $\psi (x,t)\ge 0$, $(x, t) \in \bar{\Omega }$.

#### Proof

Let $(\hat{x},\hat{t}) \in \bar{\Omega }$ be given and assume that $\psi (\hat{x},\hat{t})=\smash {\min _{\bar{\Omega }}}\psi (x,t)<0$. From the hypothesis, $(\hat{x},\hat{t}) \notin \partial \Omega$ and from the derivative tests, $\psi _{x}(\hat{x},\hat{t})=0$ and $\psi _{xx}(\hat{x},\hat{t}) > 0$.

#### Case 1

On (0, 1] by (3), $$L_{\varepsilon }\psi (\hat{x},\hat{t})= u_{t} -\varepsilon \psi _{xx}+p(\hat{x})\psi (\hat{x},\hat{t}) < \ 0$$.

#### Case 2

On (1, 2) by (3), $$L_{\varepsilon }\psi (\hat{x},\hat{t})=u_{t}-\varepsilon \psi _{xx}+p(\hat{x})\psi (\hat{x},\hat{t})+q(\hat{x})\psi (\hat{x}-1,\hat{t}) \le -\varepsilon \psi _{xx}(\hat{x},\hat{t})+2\alpha \psi (\hat{x},\hat{t}) < \ 0.$$

From the two cases, we see that $L_{\varepsilon }\psi (\hat{x},\hat{t})< 0$, which is inconsistent with the hypothesis and proves that our assumption is incorrect. As a result, $\psi (\hat{x},\hat{t})\ge 0$, which gives $\psi (x,t)\ge 0$, $(x,t) \in \bar{\Omega }$. $\square$

#### Lemma 2

Let *u*(*x*, *t*) be the solution of Eq. (1). Then, we can estimated it as

$$\begin{aligned} |u(x,t)|\le \max \left\{ \Vert u\Vert _{\partial \Omega }, \frac{1}{2\beta }\Vert L_{\varepsilon }u\Vert \right\} , \end{aligned}$$

where $$\Vert u\Vert _{\partial \Omega }=\max \{|u(0,t)|, |u(2,t)|\}$$.

#### Proof

Define barrier functions as $\omega ^{\pm }(x,t)=\max \left\{ \Vert u\Vert _{\partial \Omega }, \frac{1}{2\beta }\Vert L_{\varepsilon }u\Vert \right\} \pm u(x,t)$. Then, we have

$$\begin{aligned}&\omega ^{\pm }(0,t)= \max \left\{ \Vert u\Vert _{\partial \Omega } , \frac{1}{2\beta }\Vert L_{\varepsilon }u\Vert \right\} \pm u(0,t) \ge 0 ,\\&\omega ^{\pm }(2,t)= \max \left\{ \Vert u\Vert _{\partial \Omega } , \frac{1}{2\beta }\Vert L_{\varepsilon }u\Vert \right\} \pm u(2,t) \ge 0. \end{aligned}$$

For $x\in (0,1]$, we have

$$\begin{aligned} L_{\varepsilon }\omega ^{\pm }(x,t)= \omega ^{\pm }_{t}-\varepsilon \omega ^{\pm }_{xx}+p(x)\omega ^{\pm }(x,t) \ge p(x) \max \left\{ \Vert u\Vert _{\partial \Omega }, \frac{1}{2\beta }\Vert f\Vert \right\} \pm f(x) \ge \ 0. \end{aligned}$$

For $x\in (1,2)$, we have

$$\begin{aligned} L_{\varepsilon }\omega ^{\pm }(x,t)=&\omega ^{\pm }_{t}-\varepsilon \omega ^{\pm }_{xx}+p(x)\omega ^{\pm }(x,t)+q(x)\omega ^{\pm }(x-1,t)\\ \ge&2\beta \max \left\{ \Vert u\Vert _{\partial \Omega }, \frac{1}{2\beta }\Vert f\Vert \right\} \pm f(x) \ge 0. \end{aligned}$$

Applying Lemma 1, $\omega ^{\pm }(x,t) \ge 0$, $(x,t)\in \bar{\Omega }$, which gives the required stability estimate. $\square$

#### Lemma 3

For $(x,t) \in \bar{\Omega }$, the derivatives of the solution of Eq. (1) can be estimated as

4 $$\begin{aligned}&\left|\frac{\partial ^{m}u}{\partial t^{m}}\right|\le C\left( \Vert u\Vert _{\partial \Omega }+\Vert f\Vert +\Vert f_{t}\Vert +\Vert f_{tt}\Vert \right) , \ \ m=0,1,2, \end{aligned}$$

5 $$\begin{aligned}&\left|\frac{\partial ^{m}u}{\partial x^{m}}\right|\le C\varepsilon ^{-m/2}\left( \Vert u\Vert _{\partial \Omega }+\Vert f\Vert +\Vert f_{t}\Vert \right) , \ \ m=0,1,2, \end{aligned}$$

6 $$\begin{aligned}&\left|\frac{\partial ^{m}u}{\partial x^{m-1}\partial t}\right|\le C\varepsilon ^{(1-m)/2}\left( \Vert u\Vert _{\partial \Omega }+\Vert f\Vert +\Vert f_{t}\Vert +\Vert f_{tt}\Vert \right) , \ \ m=2,3. \end{aligned}$$

#### Proof

In Eq. (4) for $m=0$, we have $|u(x,t)|\le C$, which implies Lemma 2. For $m=1$, rearranging (1) gives

7 $$\begin{aligned} u_{t}=\varepsilon u_{xx}-p(x)u(x,t)-q(x)u(x-1,t)+f(x). \end{aligned}$$

As described by [30], if we assume that all of the data considered in (1) are zero, we have $u(x,0)=u_{0}=0$, $u(x,t)=\gamma (x,t)=0$, $u(2,t)=\zeta (t)=0$, so that (7) becomes

8 $$\begin{aligned} u_{t}=\varepsilon u_{xx}-p(x)u(x,0)-q(x)u(x-1,0)+f(x). \end{aligned}$$

For $(x,t)\in \Omega ^{-}$, we have $u(x-1,0)=\gamma (x-1,0)=0$ and for $(x,t)\in \Omega ^{+}$, we have $u(x-1,0)=\zeta (0)=0$. Hence, (8) can be reduced to $u_{t}(x,0)=f(x)$ and since *f* is smooth function, we have $|u(x,0)|\le C$, $x\in \partial \Gamma$, which gives

9 $$\begin{aligned} |u_{t}|\le C\left( \Vert u\Vert _{\partial \Omega }+\Vert f\Vert +\Vert f_{t}\Vert \right) \ \ \ {\text {o}n} \ \ \bar{\Omega }. \end{aligned}$$

Similarly, for $m=2$, assuming that the initial and boundary values considered in (1) are identically zero and differentiating with respect to *t*, we get $u_{tt}-\varepsilon u_{xxt}+p(x)u_{t}(x,t)+q(x)u_{t}(x-1,t)=f_{t}(x)$ and along $t=0$, it becomes

10 $$\begin{aligned} u_{tt}(x,0)-\varepsilon u_{xxt}(x,0)+p(x)u_{t}(x,0)+q(x)u_{t}(x-1,0)=f_{t}(x). \end{aligned}$$

Since $u_{xxt}(x,0)=f_{xx}(x)$ and $u_{t}(x-1,0)=0$, (10) becomes $u_{tt}(x,0)=\varepsilon f_{xx}(x)-p(x)f(x)+f_{t}(x)$, which implies that $|u_{tt}(x,0)|\ \le \ C$ on $\partial \Omega$ as *f* is smooth function. Hence, we have

$$\begin{aligned} |u_{tt}(x,t)|\le C\left( \Vert u\Vert _{\partial \Omega }+\Vert f\Vert +\Vert f_{t}\Vert +\Vert f_{tt}\Vert \right) \ \ {\text {o}n} \ \ \bar{\Omega }. \end{aligned}$$

Analogously, we can bound the derivatives of the solution in the spatial variable. That is, for $m=0$, we obtain the stability estimate as in Lemma 2. For $m=1$, let $x \in \Gamma$ and consider a neighborhood $I=(a, a+\sqrt{\varepsilon })$, $\forall x \in I$. Then, applying the mean value theorem for some $a\in \bar{I}$ and $t\in (0,T]$, we can get

11 $$\begin{aligned} |u_{x}(a,t)|=\sqrt{\varepsilon }|u(a+\sqrt{\varepsilon },t)-u(a,t)\sqrt{\varepsilon }|\le C\varepsilon ^{\frac{-1}{2}}\Vert u\Vert . \end{aligned}$$

Now, for any *x* in $\bar{I}$, we can get

$$\begin{aligned} |u_{x}(x,t)|=&|u_{x}(a,t)+\varepsilon ^{-1}\int _{a}^{x}(u_{t}(s,t)+p(s)u(s,t)+q(s)u(s-1,t)-f(s))ds|\\ \le&|u_{x}(a,t)|+C\varepsilon ^{-1}\left( \Vert u\Vert _{\partial \Omega }+\Vert f\Vert +\Vert f_{t}\Vert \right) . \end{aligned}$$

Using (11), we can get $|u_{x}(x,t)|\le C\varepsilon ^{\frac{-1}{2}}\left( \Vert u\Vert _{\partial \Omega }+\Vert f\Vert +\Vert f_{t}\Vert \right)$. Rearranging the terms in (1), we ge $u_{xx}=\varepsilon ^{-1}\left( u_{t}+p(x)u(x,t)+q(x) u(x-1,t)-f(x)\right)$. From this result and (9), it is possible to write the bound of $u_{xx}(x,t)$ as

$$\begin{aligned} |u_{xx}(x,t)|\le C\varepsilon ^{-1}\left( \Vert u\Vert _{\partial \Omega }+\Vert f\Vert +\Vert f_{t}\Vert \right) . \end{aligned}$$

In a similar procedure, the estimation in (6) can be confirmed and for more detail, we refer [31]. $\square$

## Discrete problem

In this section, we develop a discrete problem for the model problem (1) in two steps, first based on the time variable and then based on the spatial variable to obtain a fully discrete problem.

### Temporal semi-discretization

Let *M* denotes the number of mesh on [0, *T*]. Then, define a uniform mesh $\Omega ^{M}_{t}$ as $\Omega ^{M}_{t}=\left\{ t_{j}=j\Delta t, \ j+1=1(1) M, \ \Delta t=\frac{T}{M}\right\}$ and let’s apply the $\theta$-method for which $\theta \in [0,1]$. For $\theta \in [0,1/2)$, we obtain explicit method and in this case the method is unstable. For $\theta =1/2$, we obtain the Crank–Nicolson method and for $\theta \in (1/2,1]$, we obtain an implicit scheme. In general the $\theta$-method is numerically stable for $\theta \in [1/2,1]$ [32]. Applying the $\theta$-method finite difference approximation on $\Omega ^{M}_{t}$, (1) becomes

12 $$\begin{aligned} \left( 1+\Delta t \theta L^{M}_{\varepsilon }\right) U^{j+1}(x)=g(x,t_{j+1}), \end{aligned}$$

where

$$\begin{aligned} \left( 1+\Delta t \theta L^{M}_{\varepsilon }\right) U^{j+1}(x) ={\left\{ \begin{array}{ll} -\varepsilon \Delta t\theta U_{xx}^{j+1}(x)+[1 + \theta \Delta t p(x)]U^{j+1}(x), \ \ x\in (0,1], \\ -\varepsilon \Delta t\theta U_{xx}^{j+1}(x)+[1+ \theta \Delta t p(x)]U^{j+1}(x) \\ + \theta \Delta t q(x) U^{j+1}(x-1), \, \ x\in (1,2) \end{array}\right. } \end{aligned}$$

and

$$\begin{aligned} g(x,t_{j+1})={\left\{ \begin{array}{ll} \varepsilon \Delta t(1-\theta ) U^{j}_{xx}(x)-[-1+(1-\theta )\Delta tp(x)] U^{j}(x)+\theta \Delta t f^{j+1}(x) \\ \ -\theta \Delta t q(x)\gamma ^{j+1}(x-1) -(1-\theta )\Delta t [q(x) \gamma ^{j}(x-1)- f^{j}(x)], x\in (0,1],\\ \varepsilon \Delta t(1-\theta ) U^{j}_{xx}(x)-[-1+(1-\theta )\Delta t p(x)]U^{j}(x) +\theta \Delta t f^{j+1}(x) \\ \ -\theta \Delta t q(x)U^{j}(x-1) +(1-\theta )\Delta t f^{j}(x), \ x\in (1,2) \end{array}\right. } \end{aligned}$$

subjected to $U^{0}(x)=u_{0}(x), \ x \in \bar{\Gamma }$, $U^{j+1}(x)=\gamma (x,t_{j+1}) , \ x \in [-1,0], \ j+1=1(1)M$ and $U^{j+1}(2, t_{j+1})=\zeta (t_{j+1}),\ j+1=1(1)M$.

#### Lemma 4

(Semi-discrete maximum principle)  Let the mesh function $\varphi ^{j+1}(x)$, $j+1=1(1)M$ satisfies $\varphi ^{j+1}(x)\ge 0$, $x\in \partial \Gamma$ and $(1+\Delta t \theta L^{M}_{\varepsilon ,x})\varphi ^{j+1}(x)\ge 0$, $x \in \Gamma$. Then $\varphi ^{j+1}(x) \ge 0$, $x \in \bar{\Gamma }$.

#### Proof

For some $\hat{x} \in \Gamma$, assume that $\varphi ^{j+1}(\hat{x})=\smash {\min _{x \in \bar{\Gamma }}}\varphi ^{j+1}(x)<0$. Then, by the extreme value properties, $\varphi ^{j+1}_{x}(\hat{x})=0$ and $\varphi ^{j+1}_{xx}(\hat{x})\ge 0$, and from the given hypothesis, $\hat{x} \notin \{0,2\}$.

**Case 1:** For $\hat{x} \in (0,1]$, we have

$$\begin{aligned} (1+\Delta t \theta L^{M}_{\varepsilon ,1})\varphi ^{j+1}(\hat{x})= \varphi ^{j+1}(\hat{x})-\varepsilon \theta \Delta t \varphi _{xx}^{j+1}(\hat{x})+\Delta t \theta p(x)\varphi ^{j+1}(\hat{x}) \le \ 0. \end{aligned}$$

**Case 2:** For $\hat{x} \in (1,2)$, we have

$$\begin{aligned} (1+\Delta t \theta L^{M}_{\varepsilon ,2})\varphi ^{j+1}(\hat{x}) \le&\varphi ^{j+1}(\hat{x})+\Delta t\theta [-\varepsilon \varphi _{xx}^{j+1}(\hat{x})+ p(\hat{x})\varphi ^{j+1}(\hat{x})+ q(\hat{x}) \varphi ^{j+1}(\hat{x})] \\ \le&\ \varphi ^{j+1}(\hat{x})-\varepsilon \Delta t\theta \varphi _{xx}^{j+1}(\hat{x})+2\alpha \Delta t \theta \varphi ^{j+1}(\hat{x}) \le \ 0. \end{aligned}$$

From these two cases we see that $(1+\Delta t \theta L^{M}_{\varepsilon })\varphi ^{j+1}(\hat{x})\le 0$, for some $\hat{x} \in \Gamma$, which contradicts the given condition. This implies that $\varphi ^{j+1}(x) \ge 0$, for all $x \in \bar{\Gamma }$.

Additionally, the maximum principle is satisfied by the operator $1+\Delta t \theta L^{M}_{\varepsilon ,x}$ and as a result, we have

13 $$\begin{aligned} \left\| \left( 1+\Delta t \theta L^{M}_{\varepsilon ,x}\right) ^{-1}\right\| \le \frac{1}{1+2\beta \theta \Delta t}. \end{aligned}$$

$$\square$$

#### Lemma 5

Let the solution to the semi-discrete problem (12) be $U^{j+1}(x)$, $j+1=1(1)M$. Then, it can be estimated as

14 $$\begin{aligned} |U^{j+1}(x)|\le \frac{\Delta t}{1+2\beta \theta \Delta t}\Vert g\Vert + \max \left\{ |U^{j+1}(0)|, |U^{j+1}(2)|\right\} . \end{aligned}$$

#### Proof

To verify this estimate, define

$$\begin{aligned} \psi _{\pm }^{j+1}(x)=\frac{\Delta t}{1+2\beta \theta \Delta t}\Vert g\Vert + \max \left\{ |U^{j+1}(0)|, |U^{j+1}(2)|\right\} \pm U^{j+1}(x). \end{aligned}$$

At $x=0$, we have $\psi _{\pm }^{j+1}(0) \ge \ \frac{\Delta t}{1+2\beta \theta \Delta t}\Vert g\Vert \ge \ 0$.

At $x=2$, we have $\psi _{\pm }^{j+1}(2) \ge \ \frac{\Delta t}{1+2\beta \theta \Delta t}\Vert g\Vert \ge \ 0$.

For $x\in (0,1]$, from (12), we have

$$\begin{aligned} (1+\Delta t\theta L_{\varepsilon ,1})\psi _{\pm }^{j+1}(x) =&-\varepsilon \theta \Delta t(\psi _{xx})_{\pm }^{j+1}(x)+[1+\theta \Delta t p(x)]\psi _{\pm }^{j+1}(x)\\ \ge&[1+\theta \Delta tp(x)] \max \left\{ |U^{j+1}(0)|, |U^{j+1}(2)|\right\} \ge \ 0. \end{aligned}$$

$$\square$$

For $x\in (1,2)$, from (12), we have

$$\begin{aligned} (1+\Delta t\theta L_{\varepsilon ,2})\psi _{\pm }^{j+1}(x) =&-\varepsilon \theta \Delta t(\psi _{xx})_{\pm }^{j+1}(x)+[1+\theta \Delta t p(x)]\psi _{\pm }^{j+1}(x) \\&+\theta \Delta tq(x)(\psi _{xx})_{\pm }^{j+1}(x-1)\\ \ge&[1+\theta \Delta t(p(x)+q(x))] \max \left\{ |U^{j+1}(0)|, |U^{j+1}(2)|\right\} \ge \ 0. \end{aligned}$$

As a result, using Lemma 4, $\psi _{\pm }^{j+1}(x)\ge 0$, which implies the required stability estimate.

#### Lemma 6

Let $\left|\frac{\partial ^{m} u(x,t)}{\partial t^{m}} \right|\le C$ for $(x,t) \in \bar{\Omega }$ and $m=0,1,2$. Then, the local error is estimated as

$$\begin{aligned} \Vert e^{j+1}\Vert \le {\left\{ \begin{array}{ll} C(\Delta t)^{2} , \ \text {if} \ \ \frac{1}{2}<\theta \le 1, \\ C(\Delta t)^{3} , \ \text {if}\ \ \theta =\frac{1}{2}. \end{array}\right. } \end{aligned}$$

#### Proof

The local truncation error can be determined by selecting an appropriate base point, where the Taylor series is expanded. The convenient base point for a fully implicit method is the point $(x_{i}, t_{j+1})$. However, the center point, $(x_{i},t_{j+\frac{1}{2}})$ is a convenient base point for any value of $\theta \in [\frac{1}{2},1)$ and using this base point, the Taylor series expansion yield

15 $$\begin{aligned} \left( 1+\Delta t \theta L^{M}_{\varepsilon ,x}\right) u^{j+1}(x)=g(x,t_{j+1})+(\frac{1}{2}-\theta )O((\Delta t)^{2})+O((\Delta t)^{3}). \end{aligned}$$

Taking the difference between (12) and (15), we obtain the local error satisfying

$$\begin{aligned}&\left( 1+\Delta t \theta L^{M}_{\varepsilon ,x}\right) e^{j+1}=\left( \frac{1}{2}-\theta \right) O((\Delta t)^{2})+O((\Delta t)^{3}),\\&\,\,\,\,\, \,\,\, e^{j+1}(0)=0, \, e^{j+1}(2)=0. \end{aligned}$$

Thus, applying Lemma 4, we can obtain the required estimate. $\square$

#### Lemma 7

Assuming that Lemma 6 holds true, the global truncation errors $E^{j+1}$ can be estimated as

16 $$\begin{aligned} \Vert E^{j+1}\Vert \le {\left\{ \begin{array}{ll} C(\Delta t), \ \text {if} \ \frac{1}{2}< \theta \le 1, \\ C(\Delta t)^{2}, \ \text {if} \ \theta =\frac{1}{2}. \end{array}\right. } \end{aligned}$$

#### Proof

By the local error estimate up to the $(j+1)^{th}$ time level for $\theta =\frac{1}{2}$, we have

$$\begin{aligned} \Vert E^{j+1}\Vert& =\left\| \sum _{k=1}^{j+1} e^{k}\right\| , \ \ (j+1)\Delta T\le T \\ \le&\Vert e^{1}\Vert +\Vert e^{2}\Vert +...+\Vert e^{j+1}\Vert \le C_{1}(j\Delta t)(\Delta t)^{2} \le C(\Delta t)^{2}. \end{aligned}$$

By a similar procedures for $\frac{1}{2}<\theta \le 1$, we have

$$\begin{aligned} \Vert E^{j+1}\Vert &=\left\| \sum _{k=1}^{j} e^{k}\right\| , \ \ j(\Delta t)\le T \\ \le&\Vert e^{1}\Vert +\Vert e^{2}\Vert +...+\Vert e^{j}\Vert \le C_{1}(j\Delta t)\Delta t \le C(\Delta t). \end{aligned}$$

$$\square$$

#### Remark 1

To obtain an improved error estimate, we decompose $U^{j+1}(x)$ as $U^{j+1}(x)=V^{j+1}(x)+W^{j+1}(x)$, where $V^{j+1}(x)$ is the smooth component satisfying the problem

$$\begin{aligned} -\varepsilon \Delta t \theta V^{j+1}_{xx}(x)&+[1+\theta \Delta t p(x)]V^{j+1}(x)\\ =&\varepsilon \Delta t(1-\theta )V^{j}_{xx}(x)+[1-(1-\theta )\Delta t p(x)]V^{j}(x)+\theta \Delta t f^{j+1}(x)\\&-\theta \Delta t q(x)\gamma ^{j+1}(x-1)-(1-\theta )\Delta t [q(x)\gamma ^{j}(x-1)-f^{j}(x)], \, x\in (0,1],\\&V^{j+1}(0)=V^{j+1}(0),\\&V^{j+1}(1)=[1+\theta \Delta tp(1)]^{-1} ([1-(1-\theta )\Delta t p(1)]V^{j}(1)+\theta \Delta t f^{j+1}(1)\\&\,\,\, \,\,\,\,\,\,\,\,\,\,-\theta \Delta t q(x)\gamma ^{j+1}(0)-(1-\theta )\Delta t [q(1)\gamma ^{j}(0)-f^{j}(1)]), \end{aligned}$$

$$\begin{aligned} -\varepsilon \Delta t \theta V^{j+1}_{xx}(x)&+[1+\theta \Delta t p(x)]V^{j+1}(x)+\theta \Delta t q(x)V^{j+1}(x-1)\\ =&\varepsilon \Delta t(1-\theta )V^{j}_{xx}(x)+[1-(1-\theta )\Delta t p(x)]V^{j}(x)+\theta \Delta t f^{j+1}(x)\\&-(1-\theta )\Delta t [q(x)V^{j}(x-1)-f^{j}(x)], \, x\in (1,2],\\&V^{j+1}(2)=V^{j+1}(2),\\&V^{j+1}(1)=[1+\theta \Delta tp(1)]^{-1} ([1-(1-\theta )\Delta t p(1)]V^{j}(1)+\theta \Delta t f^{j+1}(1)\\&\,\,\, \,\,\,\,\,\,\,\,\,\,-(1-\theta )\Delta t [q(1)V^{j}(0)-f^{j}(1)]), \end{aligned}$$

and $W^{j+1}(x)$ is the singular component satisfying the problem

$$\begin{aligned}&-\varepsilon \Delta t \theta W_{xx}^{j+1}(x)+[1+\theta \Delta t p(x)]W^{j+1}(x)=0, \, x\in (0,1]\\&-\varepsilon \Delta t \theta W_{xx}^{j+1}(x)+[1+\theta \Delta t p(x)]W^{j+1}(x)+\theta \Delta t q(x)W^{j+1}(x-1), \,\, x\in (1,2),\\&W^{j+1}(0)=U^{j+1}(0)-V^{j+1}(0), \,\, W^{j+1}(2)=U^{j+1}(2)-V^{j+1}(2). \end{aligned}$$

#### Lemma 8

The derivatives of $U^{j+1}(x),\, j+1=1(1)M$ and its components can be estimated as

$$\begin{aligned}&\left|\frac{d^{m}U^{j+1}(x)}{dx^{m}}\right|\le {\left\{ \begin{array}{ll} C(1+\varepsilon ^{-\frac{m}{2}}a_{1}(x,\beta )),\, 0\le x\le 1, \\ C(1+\varepsilon ^{-\frac{m}{2}}a_{2}(x,\beta )),\, 1\le x \le 2, \end{array}\right. }\\&\left|\frac{d^{m}V^{j+1}(x)}{dx^{m}}\right|\le {\left\{ \begin{array}{ll} C(1+\varepsilon ^{\frac{-(m-2)}{2}}a_{1}(x, \beta )), \ \ 0\le x\le 1, \\ C(1+\varepsilon ^{\frac{-(m-2)}{2}}a_{2}(x,\beta ), \ \ \ 1\le x\le 2, \end{array}\right. }\\&\left|\frac{d^{m}W^{j+1}(x)}{dx^{m}}\right|\le {\left\{ \begin{array}{ll} C\varepsilon ^{\frac{-m}{2}}a_{1}(x,\beta ), \ 0\le x \le 1, \\ C\varepsilon ^{\frac{-m}{2}}a_{2}(x, \beta ), \ 1 \le x \le 2, \end{array}\right. } \end{aligned}$$

where $$a_{1}(x, \beta )=e^{-\sqrt{\frac{\beta }{\varepsilon }}x}+e^{-\sqrt{\frac{\beta }{\varepsilon }}(1-x)}$$, $$a_{2}(x, \beta )=e^{-\sqrt{\frac{\beta }{\varepsilon }}(x-1)}+e^{-\sqrt{\frac{\beta }{\varepsilon }}(2-x)}$$ and $$m=0, 1, 2, 3$$ $$.$$

#### Proof

For the proof, we refer [27, 33]. $\square$

### Spatial discretization

Here, we start by splitting the spatial domain [0, 2] into two sub-intervals [0, 1] and (1, 2]. For *N* mesh numbers, consider $\bar{\Omega }^{N}_{x}$ as a piece-wise uniform spatial mesh. Then, each sub-intervals can be further divided as $[0,1]=[0,\sigma ]\cup (\sigma , 1-\sigma ]\cup (1-\sigma , 1]$ and $(1,2]=(1,1+\sigma ]\cup (1+\sigma ,2-\sigma ]\cup (2-\sigma ,2]$, Where $\sigma$ is a transition parameter, which separates the non-uniform into uniform meshes and given as $\sigma =\min \left\{ \frac{1}{4},2\sqrt{\frac{\varepsilon }{\beta }}\ln N\right\}$. We consider $\frac{N}{8}$ mesh numbers on each of the fine mesh regions $[0,\sigma ]$, $(1-\sigma ,1)$ , $(1,1+\sigma )$ and $(2-\sigma ,2)$, and $\frac{N}{4}$ mesh numbers on each of the coarse mesh regions $(\sigma ,1-\sigma ]$ and $(1+\sigma ,2-\sigma ]$. The nodal mesh points are $x_{i}=x_{i-1}+h_{i} , \ i=1,2, ... , N$ , where the mesh interval $h_{i}$’s are given by

$$\begin{aligned} h_{i}={\left\{ \begin{array}{ll} \frac{8\sigma }{N} ,\ \ \ \ \ \ i=1,2,..., N/8 , 3N/8+1,...,N/2 , N/2+1,...,5N/8 , 7N/8+1,...,N,\\ \frac{4(1-2\sigma )}{N} , \ \ \ \ i=N/8+1,...,3N/8 , 5N/8+1,...,7N/8. \end{array}\right. } \end{aligned}$$

Now, let’s define the standard finite difference operators as

$$\begin{aligned}&D^{+}U^{j+1}(x_{i})=\frac{U^{j+1}_{i+1}-U^{j+1}_{i}}{h_{i+1}}, \ \ D^{-}U^{j+1}(x_{i})=\frac{U^{j+1}_{i}-U^{j+1}_{i-1}}{h_{i}},\\&D^{+}D^{-}U^{j+1}(x_{i})=\frac{2}{h_{i}+h_{i+1}}\left[ D^{+}U^{j+1}(x_{i})-D^{-}U^{j+1}(x_{i})\right]. \end{aligned}$$

Substituting the differential operator in Eq. (12) by the standard finite difference operators, we obtain a fully-discretized problem as

17 $$\begin{aligned} L^{N,M}_{\varepsilon }U_{i}^{j+1}=g(x_{i},t_{j+1}) ,\, i=1,2,...,N-1,\, j+1=1,2,...,M, \end{aligned}$$

where

$$\begin{aligned} L^{N,M}_{\varepsilon }U_{i}^{j+1}={\left\{ \begin{array}{ll} -\varepsilon \Delta t\theta D^{+}D^{-}U^{j+1}_{i}+(1+\Delta t \theta p_{i})U^{j+1}_{i},\,\, i=1, 2,..., N/2, \\ -\varepsilon \Delta t\theta D^{+}D^{-}U^{j+1}_{i}+(1+\Delta t\theta p_{i})U^{j+1}_{i}+\Delta t \theta q_{i}U^{j+1}_{N/2-1},\\ \qquad \, i=N/2+1, N/2+2,...,N-1 \end{array}\right. } \end{aligned}$$

and

$$\begin{aligned} g(x_{i},t_{j+1})={\left\{ \begin{array}{ll} &{}\Delta t(1-\theta ) [\varepsilon D^{+}D^{-}U^{j}_{i}-p_{i}U^{j}_{i} -q_{i}\gamma ^{j}(x_{i}-1) +f(x_{i})] \\ &{} -\theta \Delta t q_{i}\gamma ^{j+1}(x_{i}-1) +\theta \Delta t f(x_{i})+U^{j}_{i}, \, \, i=1(1)N/2 \\ &{}\Delta t(1-\theta )[\varepsilon D^{+}D^{-}U^{j}_{i}- p_{i}U^{j}_{i}- q_{i}U^{j}_{N/2-1}+f(x_{i})]\\ &{} \ +U^{j}_{i} +\Delta t \theta f(x_{i}), \,\,i=N/2+1(1)N-1. \end{array}\right. } \end{aligned}$$

From Eq. (17), we obtain a system of equations of the form

$$\begin{aligned} r_{i}^{-}U_{i-1}^{j+1}+r_{i}^{0}U_{i}^{j+1}+r_{i}^{+}U_{i+1}^{j+1}=G_{i}, \, \, i=1(1)N/2, \end{aligned}$$

where $$\begin{aligned}&r_{i}^{-}=\frac{-2\varepsilon \theta }{h_{i}(h_{i}+h_{i+1})}, \, r_{i}^{0}=\frac{2\varepsilon \theta }{h_{i+1}(h_{i}+h_{i+1})}+\frac{2\varepsilon \theta }{h_{i}(h_{i}+h_{i+1})}+\frac{1}{\Delta t}\\&+\theta p_{i},\, r_{i}^{+}=\frac{-2\varepsilon \theta }{h_{i+1}(h_{i}+h_{i+1})},\, G_{i}=(1-\theta ) [\varepsilon D^{+}_{x}D^{-}_{x}U^{j}_{i}-p_{i}U^{j}_{i} -q_{i}\gamma^{j}\\&(x_{i}-1) +f^{j}(x_{i})] -\theta q_{i}\gamma ^{j+1}(x_{i}-1) +\theta f^{j+1}(x_{i})+(1/\Delta t)U^{j}_{i}.\end{aligned}$$

Similarly, we can obtain

$$\begin{aligned} r_{i}^{-}U_{i-1}^{j+1}+r_{i}^{0}U_{i}^{j+1}+r_{i}^{+}U_{i+1}^{j+1}= G^{*}_{i}, \, \, i=N/2+1 (1) N, \end{aligned}$$

where $r_{i}^{-}$, $r_{i}^{0}$ and $r_{i}^{+}$ are provided as above and $G^{*}_{i}=(1-\theta )[\varepsilon D^{+}D^{-}U^{j}_{i}- p_{i}U^{j}_{i}- q_{i}U^{j}_{N/2-1}+f^{j}(x_{i})] +(1/\Delta t)U^{j}_{i} +\theta f^{j+1}(x_{i})$.

#### Lemma 9

(Discrete Maximum Principle) Let $\varphi ^{j+1}_{i}$ be a given mesh function, which satisfies $\varphi ^{j+1}_{0}\ge 0$ and $\varphi ^{j+1}_{N}\ge 0$, $j+1=1(1)M$. If $L^{N,M}_{\varepsilon } \varphi ^{j+1}_{i}\ge 0$, $i=1(1)N-1$, then $\varphi ^{j+1}_{i}\ge 0$, $i=0(1)N$.

#### Proof

Assume that $\varphi ^{j+1}_{\iota }=\smash {\displaystyle \min _{i=0,1,...,N}}\varphi ^{j+1}_{i} <0$ for some $\iota \in \{0,1,...,N\}$ .

#### Case 1

For $i=1(1)\frac{N}{2}$, we have

$$\begin{aligned} L^{N}_{\varepsilon ,1}\varphi ^{j+1}_{\iota } = \ - \varepsilon \Delta t\theta D^{+}D^{-}\varphi ^{j+1}(x_{\iota })+(1/\Delta t+\theta p(x_{\iota }))\varphi ^{j+1}(x_{\iota }) < \ 0. \end{aligned}$$

#### Case 2

For $i=\frac{N}{2}+1(1)N-1$, we have

$$\begin{aligned} L^{N}_{\varepsilon ,2 }\varphi ^{j+1}_{\iota } =&- \varepsilon \theta D^{+}D^{-}\varphi ^{j+1}(x_{\iota })+(1/\Delta t+\theta p(x_{\iota }))\varphi ^{j+1}(x_{\iota }) +\theta q(x_{\iota })\varphi ^{j+1}(x_{\iota }-1)\\ \le&- \varepsilon \theta D^{+}D^{-}\varphi ^{j+1}(x_{\iota })+[1/\Delta t+\theta p(x_{\iota }) +\theta q(x_{\iota })]\varphi ^{j+1}(x_{\iota }) < \ 0. \end{aligned}$$

From the two cases, we obtained a contradiction to the given hypothesis, which implies that $\varphi ^{j+1}_{i}\ge 0$ for all $i=0(1)N$, $j+1=1(1)M$. $\square$

#### Lemma 10

(Stability of the discrete problem)   Let $U^{j+1}_{i}$, $i=0(1)N, \, j+1=1(1)M$ be a solution of the fully-discrete problem (17). Then, it is estimated as

$$\begin{aligned} |U^{j+1}_{i}|\le \frac{\Delta t\Vert g\Vert }{1+2\beta \theta \Delta t}+ \max \left\{ |U^{j+1}_{0}|, \ |U^{j+1}_{N}|\right\} . \end{aligned}$$

#### Proof

To confirm this estimate, define two barrier functions as

$$\begin{aligned} (\vartheta ^{j+1}_{i})^{\pm }= \frac{\Delta t\Vert g\Vert }{1+2\beta \theta \Delta t}+ \max \left\{ |U^{j+1}_{0}|, \ |U^{j+1}_{N}|\right\} \pm U^{j+1}_{i}. \end{aligned}$$

The estimate holds true for $i\in \{0,N\}$, and for other values of *i*, we examine the two cases below.

#### Case 1

For $i=1(1)\frac{N}{2}$, we get

$$\begin{aligned} L^{N,M}_{1}(\vartheta ^{j+1}_{i})^{\pm }=&-\varepsilon \theta D^{+}D^{-}(\vartheta ^{j+1}_{i})^{\pm }+(1/\Delta t+\theta p_{i})(\vartheta ^{j+1}_{i})^{\pm }\\ \ge&(1/\Delta t+\theta p_{i})\left( \frac{\Delta t\Vert g\Vert }{1+2\beta \theta \Delta t}+\max \left\{ |U^{j+1}_{0}|, \ |U^{j+1}_{N}|\right\} \right) \pm g(x_{i},t_{j+1})\\ \ge&(1/\Delta t+\theta p_{i})\max \left\{ |U^{j+1}_{0}|, \ |U^{j+1}_{N}|\right\} \ge \ 0. \end{aligned}$$

#### Case 2

For $i=\frac{N}{2}+1(1)N-1$, we get

$$\begin{aligned} L^{N}_{2}(\vartheta ^{j+1}_{i})^{\pm }=&-\varepsilon \theta D^{+}D^{-}(\vartheta ^{j+1}_{i})^{\pm }+(1/\Delta t+\theta p(x_{i}))(\vartheta ^{j+1}_{i})^{\pm }+\theta q(x_{i})(\vartheta ^{j+1}_{i-\frac{N}{2}})^{\pm }\\ \ge&(1/\Delta t+\theta p_{i}+\theta q_{i})\left( \frac{\Delta t\Vert g\Vert }{1+2\beta \theta \Delta t}+\max \left\{ |U^{j+1}_{0}|, \ |U^{j+1}_{N}|\right\} \right) \pm g(x_{i},t_{j+1})\\ \ge&(1/\Delta t+\theta p_{i}+\theta q_{i})\max \left\{ |U^{j+1}_{0}|, \ |U^{j+1}_{N}|\right\} \ge \ 0. \end{aligned}$$

So, applying Lemma 9, the required stability estimate can be obtained. $\square$

#### Remark 2

To obtain an improved error estimate for the discrete scheme, the solution $U^{j+1}_{i}$ can be decomposed into its regular component $V^{j+1}_{i}$ and singular component $W^{j+1}_{i}$ as $U^{j+1}_{i}=V^{j+1}_{i}+W^{j+1}_{i}$. The regular component satisfies he problem

$$\begin{aligned} L^{N, M}_{\varepsilon }V_{i}^{j+1}=g^{j+1}(x_{i}), \, i=1(1)N-1,\, j+1=1(1)M, \end{aligned}$$

where

$$\begin{aligned}&L^{N, M}_{\varepsilon }V^{j+1}_{i}={\left\{ \begin{array}{ll} -\varepsilon \theta D^{+}D^{-}V^{j+1}_{i}+(1/\Delta t+\theta p_{i})V^{j+1}_{i}, \, i=1(1)\frac{N}{2},\\ -\varepsilon \theta D^{+}D^{-}V^{j+1}_{i}+(1/\Delta t+\theta p_{i})V^{j+1}_{i}+\theta q(x_{i})V^{j+1}_{\frac{N}{2}-1}, \\ \qquad \qquad i=\frac{N}{2}+1(1)N-1, \end{array}\right. } \\&\ \ \ \ \ V^{j+1}_{0}=V^{j+1}(0), \, V^{j+1}_{\frac{N}{2}-1}=V^{j+1}(1^{-}), \, V^{j+1}_{\frac{N}{2}+1}=V^{j+1}(1^{+}), \, V^{j+1}_{N}=V^{j+1}(2). \end{aligned}$$

And the singular component, $W^{j+1}_{i}$ satisfies the problem

$$\begin{aligned}&L^{N, M}_{\varepsilon }W^{j+1}_{i}={\left\{ \begin{array}{ll} -\varepsilon \theta D^{+}D^{-}W^{j+1}_{i}+(1/\Delta t+\theta p_{i})W^{j+1}_{i}=0,\, i=1(1)N/2,\\ -\varepsilon \theta D^{+}D^{-}W^{j+1}_{i}+(1/\Delta t+\theta p_{i})W^{j+1}_{i}+\theta q_{i}W^{j+1}_{\frac{N}{2}-1}=0,\\ \, \, \, \, \, i=\frac{N}{2}+1(1)N-1, \end{array}\right. }\\&\ \ \ \ W^{j+1}_{0}=W^{j+1}(0) , \, W^{j+1}_{N}=W^{j+1}(2). \end{aligned}$$

#### Theorem 1

Suppose that $U^{j+1}(x_{i})$ is the solution of (12) and $U^{j+1}_{i}$ is the solution of (17). Then, the local truncation error at the $(j+1)^{th}$ time level is given by

$$\begin{aligned} |U^{j+1}(x_{i})-U^{j+1}_{i}|\le C(N^{-1}\ln N)^{2}. \end{aligned}$$

#### Proof

The local truncation error at each mesh point $(x_{i}, t_{j+1})$ is given as $e^{j+1}_{i}= U^{j+1}(x_{i})-U^{j+1}_{i}$. So, we can treat the error estimates in the regular and singular components separately. Using the classical argument for $i=1(1)\frac{N}{2}-1$, the local error of the regular component is

$$\begin{aligned} L^{N,M}_{\varepsilon }(V^{j+1}-V^{j+1}(x_{i}))=-\varepsilon \theta \left( D^{+}D^{-}-\frac{d^{2}}{dx^{2}}\right) V^{j+1}(x_{i}). \end{aligned}$$

Using the results in chapters 4 and 6 of [34], we obtain

18 $$\begin{aligned} |L^{N,M}_{\varepsilon }(V^{j+1}_{i}-V^{j+1}(x_{i}))|\le {\left\{ \begin{array}{ll} C\varepsilon \theta (x_{i+1}-x_{i-1})|V^{j+1}(x_{i})|_{3}, \ \ x_{i}\in (\sigma , 1-\sigma ),\\ C\varepsilon \theta (x_{i}-x_{i-1})^{2}|V^{j+1}(x_{i})|_{4}, \ \ { otherwise}. \end{array}\right. } \end{aligned}$$

And for $m=0(1)4$, the regular component satisfies

19 $$\begin{aligned} |V^{(m)}(x_{i},t_{j+1})|\le C\left( 1+\varepsilon ^{\frac{-(m-2)}{2}}\right) , \ x_{i}\in [0,1], \end{aligned}$$

which implies that $${\left\{ \begin{array}{ll} |V'''(x_{i},t_{j+1})|\le C(1+\varepsilon ^{-1/2}) , \\ |V^{(4)}(x_{i},t_{j+1})|\le C(1+\varepsilon ^{-1}). \end{array}\right. }$$

Since $x_{i+1}-x_{i-1}\le 2/N$, (18) and (19) yield

$$\begin{aligned} |L^{N,M}_{\varepsilon }(V^{j+1}_{i}-V^{j+1}(x_{i}))|\le {\left\{ \begin{array}{ll} C N^{-1}\varepsilon (1+\varepsilon ^{-1/2}), \ \ x_{i}\in (\sigma , 1-\sigma ),\\ C N^{-2}\varepsilon (1+\varepsilon ^{-1}), \ \ { otherwise}. \end{array}\right. } \end{aligned}$$

Taking the smaller order of $\varepsilon$, we get $$|L^{N,M}_{\varepsilon }(V^{j+1}_{i}-V^{j+1}(x_{i}))|\le {\left\{ \begin{array}{ll} C N^{-1}\varepsilon ^{1/2}, \ \ x_{i}\in (\sigma , 1-\sigma ),\\ C N^{-2}, \ \ otherwise. \end{array}\right. }$$

Now, introduce $$\psi (x_{i}) = CN^{-2}+CN^{-2}\frac{\sigma }{\sqrt{\varepsilon }}{\left\{ \begin{array}{ll} x_{i}/\sigma , \ \ 0\le x_{i}\le \sigma , \\ 1, \ \ \sigma \le x_{i}\le 1-\sigma ,\\ (1-x_{i})/\sigma , \ \ 1-\sigma \le x_{i}\le 1, \end{array}\right. }$$ which provide $\psi (x_{i}) = CN^{-2}+CN^{-2}\frac{\sigma }{\sqrt{\varepsilon }}(1)$, for $x_{i}\in (\sigma , 1-\sigma )$. Since $\sigma =2\sqrt{\frac{\varepsilon }{\beta }}\ln N$, we can obtain $\psi (x_{i}) \le CN^{-2}\ln N$. Consequently, we have $L^{N,M}_{\varepsilon }\left( \psi (x_{i})\pm (V^{j+1}_{i}-V^{j+1}(x_{i}))\right) \ge 0$ and $$L^{N,M}_{\varepsilon }\psi (x_{i})\ge {\left\{ \begin{array}{ll} C N^{-1}\varepsilon ^{1/2}, \ \ x_{i}\in (\sigma , 1-\sigma ),\\ C N^{-2}, \ \ otherwise. \end{array}\right. }$$

Applying Lemma 9, we obtain $|V^{j+1}_{i}-V^{j+1}(x_{i})|\le \psi (x_{i})\le CN^{-2}\ln N$. Following similar procedure for $i=\frac{N}{2}+1, \frac{N}{2}+2, ..., N-1$, we can obtain $|V^{j+1}_{i}-V^{j+1}(x_{i})|\le \psi (x_{i})\le CN^{-1}$ and combining these outcomes gives

20 $$\begin{aligned} |V^{j+1}_{i}-V^{j+1}(x_{i})|\le CN^{-2}\ln N. \end{aligned}$$

For the singular component, the error is estimated considering the transition parameter $\sigma =\frac{1}{4}$ and $\sigma =2\sqrt{\frac{\varepsilon }{\beta }}\ln N$ separately. That is, for the case $\sigma =\frac{1}{4}$, we have a uniform mesh and $2\sqrt{\frac{\varepsilon }{\beta }}\ln N \ge \frac{1}{4}$, so that $\varepsilon ^{-1/2}\le C \ln N$ . By the classical argument we have $|L^{N,M}_{\varepsilon }(W^{j+1}_{i}-W^{j+1}(x_{i}))|\le C\theta (x_{i}-x_{i-1})^{2} \varepsilon |W(x_{i})|_{4}$. Since $|W^{j+1}(x_{i})|_{4}\le C\varepsilon ^{-2}$ and $x_{i}-x_{i-1}\le N^{-1}$, we have $|L^{N,M}_{\varepsilon }(W^{j+1}_{i}-W^{j+1}(x_{i}))|\le C(N^{-1}\ln N)^{2}$. Consequently, by using the maximum principle, we have

21 $$\begin{aligned} |(W^{j+1}_{i}-W^{j+1}(x_{i}))|\le C(N^{-1}\ln N)^{2} , \ \ \ \forall x_{i}\in [0,\sigma ]. \end{aligned}$$

By a similar argument on $(1-\sigma , 1)$, we obtain the same estimate as in (21). For the case $\sigma \le \frac{1}{4}$, a piece-wise uniform meshes exist with $\frac{4(1-2\sigma )}{N}$ mesh size in $[\sigma ,1-\sigma ]$ and $\frac{8\sigma }{N}$ mesh size in each of $[0,\sigma ]$ and $[1-\sigma ,1]$. Thus, to estimate $|W^{j+1}_{i}-W^{j+1}(x_{i})|$, different arguments can be considered depending on the mesh spacing. For $x_{i}$ in the sub-intervals $[0,\sigma ]$ and $[1-\sigma ,1]$ the classical argument gives $|L^{N,M}_{\varepsilon }(W_{i}^{j+1}-W^{j+1}(x_{i}))|\le C\varepsilon \theta (x_{i}-x_{i-1})^{2}|W^{j+1}(x_{i})|_{4}$ . Since $x_{i}-x_{i-1}=\frac{8\sigma }{N}$ and $|W^{j+1}(x_{i}|_{4}\le C\varepsilon ^{-2}$, we have $\vert L^{N,M}_{\varepsilon }(W_{i}^{j+1}-W^{j+1}(x_{i}))|\le C\varepsilon \theta \left( \frac{8\sigma }{N}\right) ^{2}C\varepsilon ^{-2}\le CN^{-2}\sigma ^{2}\varepsilon ^{-1}$. But $\sigma =2\sqrt{\frac{\varepsilon }{\beta }}\ln N$ and hence $|L^{N,M}_{\varepsilon }(W_{i}^{j+1}-W^{j+1}(x_{i}))|\le CN^{-2}\sigma ^{2}\varepsilon ^{-1}\le C(N^{-1}\ln N)^{2}$. By the maximum principle, we have $|(W^{j+1}_{i}-W^{j+1}(x_{i}))|\le C(N^{-1}\ln N)^{2}, \ \ x_{i}\in [0,\sigma ]\cup [1-\sigma , 1]$. On the sub-interval $[\sigma ,1-\sigma ]$, the local truncation error of the singular component is

$$\begin{aligned} |L^{N,M}_{\varepsilon } (W^{j+1}_{i}-W^{j+1}(x_{i}))|\le \varepsilon \theta |\left( D^{+}D^{-}-\frac{d^{2}}{dx^{2}}\right) W^{j+1}(x_{i})|. \end{aligned}$$

However, $|D^{+}D^{-}W^{j+1}(x_{i})|\le \smash {\displaystyle \max _{x\in [x_{i-1},\, x_{i+1}]}}|W''(x_{i},t_{j+1})|$, and as a result, we have

$$\begin{aligned} |L^{N,M}_{\varepsilon } (W^{j+1}_{i}-W^{j+1}(x_{i}))|=&\varepsilon \theta |\left( D^{+}D^{-}-\frac{d^{2}}{dx^{2}}\right) W^{j+1}(x_{i})|\\ \le&\varepsilon \theta \left( |D^{+}D^{-}W^{j+1}(x_{i})|+\left|\frac{d^{2}W^{j+1}(x_{i})}{dx^{2}}\right|\right) \\ \le&2\varepsilon \theta \smash {\displaystyle \max _{x\in [x_{i-1},\, x_{i+1}]}}|W''(x_{i},t_{j+1})|\le \ 2\varepsilon \theta |W^{j+1}({x_{i}})|_{2}. \end{aligned}$$

Using the estimate for $W''(x_{i},t_{j+1})$, we have

$$\begin{aligned} |L^{N,M}_{\varepsilon } (W^{j+1}_{i}-W^{j+1}(x_{i}))|\le \ 2\varepsilon \theta |W^{j+1}({x_{i}})|_{2} \le&\ 2\varepsilon \theta C\varepsilon ^{-1} e^{-x_{i-1}\sqrt{\frac{\beta }{\varepsilon }}} , \ \ \ x_{i-1}< \sigma \\ \le&\ Ce^{-\sqrt{\frac{\beta }{\varepsilon }}(\sigma -4\sigma /N)} \\ \le&\ CN^{-2}, \ \sigma =2\sqrt{\frac{\varepsilon }{\beta }}\ln N, \end{aligned}$$

which implies that $|L^{N,M}_{\varepsilon } (W^{j+1}_{i}-W^{j+1}(x_{i}))|\le CN^{-2}\le C(N^{-1}\ln N)^{2}, \ \ \ x_{i}\in (\sigma ,1-\sigma )$. So, we have $|(W^{j+1}_{i}-W^{j+1}(x_{i}))|\le C(N^{-1}\ln N)^{2}$ on $[\sigma , 1-\sigma ]$. Combining the aforementioned findings for the singular components on (0, 1] with analogous procedures and results on the interval (1, 2), we can obtain

22 $$\begin{aligned} |W^{j+1}_{i}-W^{j+1}(x_{i})|\le C(N^{-1}\ln N)^{2}. \end{aligned}$$

Thus, from the results in (20) and (22), we have

23 $$\begin{aligned} |U^{j+1}_{i}-U^{j+1}(x_{i})|\le C(N^{-1}\ln N)^{2}, \end{aligned}$$

which is the error estimate at the $(j+1)^{th}$ time level. $\square$

#### Theorem 2

Let *u*(*x*, *t*) be the solution of the continuous problem (1) and $U^{j+1}_{i}$ be the solution of the discrete problem (17). Then, the uniform error estimate given as

$$\begin{aligned} \smash {\displaystyle \max _{i=0(1)N,\,j+1=1(1)M}}|u(x_{i},t_{j+1})-U^{j+1}_{i}|\le {\left\{ \begin{array}{ll} C(\Delta t+(N^{-1}\ln N)^{2}), \ \ \frac{1}{2}<\theta \le 1,\\ C((\Delta t)^{2}+(N^{-1}\ln N)^{2}), \ \ \ \theta =\frac{1}{2}. \end{array}\right. } \end{aligned}$$

#### Proof

From Lemma 7 and Theorem 1, we have

$$\begin{aligned} |u(x_{i},t_{j+1})-U^{j+1}_{i}|\le \,&|u(x_{i},t_{j+1})-U^{j+1}(x_{i})|+|U^{j+1}(x_{i})-U^{j+1}_{i}|\\ \le&\ {\left\{ \begin{array}{ll} C(\Delta t+(N^{-1}\ln N)^{2}), \ \ \frac{1}{2}<\theta \le 1\\ C((\Delta t)^{2}+(N^{-1}\ln N)^{2}), \ \ \theta =\frac{1}{2} \end{array}\right. } \end{aligned}$$

by which we achieved the uniform error estimate for $i=0(1)N, j+1=1(1)M$. $\square$

## Numerical experiments and discussions

In this section, to demonstrate the validity and applicability of the obtained numerical scheme, we carry out numerical experiments for the problem under consideration. If the exact solution of a problem is known, we compute the maximum absolute error as the difference between the exact solution and the numerical solution obtained using the developed method. However, if the exact solution is not known, the maximum absolute error is computed using the double mesh principle [35] as $E^{N,M}_{\varepsilon }=\smash {\displaystyle \max _{0\le i\le N, 0\le j\le M}}|U^{N,M}(x_{i},t_{j})-U^{2N,2M}(x_{i},t_{j})|$, where $U^{2N,2M}(x_{i},t_{j})$ is a numerical solution obtained by doubling both the spatial and temporal mesh numbers, fixing the transition parameter. And the parameter-uniform absolute error is obtained by $E^{N,M}=\smash {\max _{\varepsilon }}(E^{N,M}_{\varepsilon })$. We compute the nodal rate of convergence as $R^{N,M}_{\varepsilon }=\frac{\log (E^{N,M}_{\varepsilon }/E_{\varepsilon }^{2N,2M})}{\log {2}}$ and the uniform convergence rate is obtained by $R^{N,M}=\frac{\log (E^{N,M}/E^{2N,2M})}{\log {2}}$.

### Example 1

Consider problem (1) with $p(x)=3$, $q(x)=-1$, $f(x)=1$, $u_{0}(x)=0$, $\gamma (x,t)=0$ and $\zeta (t)=0$ [22].

### Example 2

Consider problem (1) with $p(x)=5$, $q(x)=-2$, $f(x)=2$, $u_{0}(x)=\sin (\pi x)$, $\gamma (x,t)=0$ and $\zeta (t)=0$ [21].

We have treated the two examples applying the numerical scheme developed in this paper using MATLAB R2019a software packages. Since the exact solutions are not known, the double mesh principle is applied and the obtained results are given in tabular and graphical forms. The maximum errors and rate of convergences are given in Table 1 for Example 1 and in Table 2 for Example 2. From these tables, we observe that for a given $\varepsilon$, increasing both time and space mesh numbers, decreases the absolute point-wise error. And by fixing the mesh numbers and decreasing the perturbation parameter results in stabled point-wise error. This indicates the $\varepsilon$-uniform convergence of the proposed numerical schemes. Table 3 shows comparisons of the developed method with other results published in literature.

**Fig. 1:**
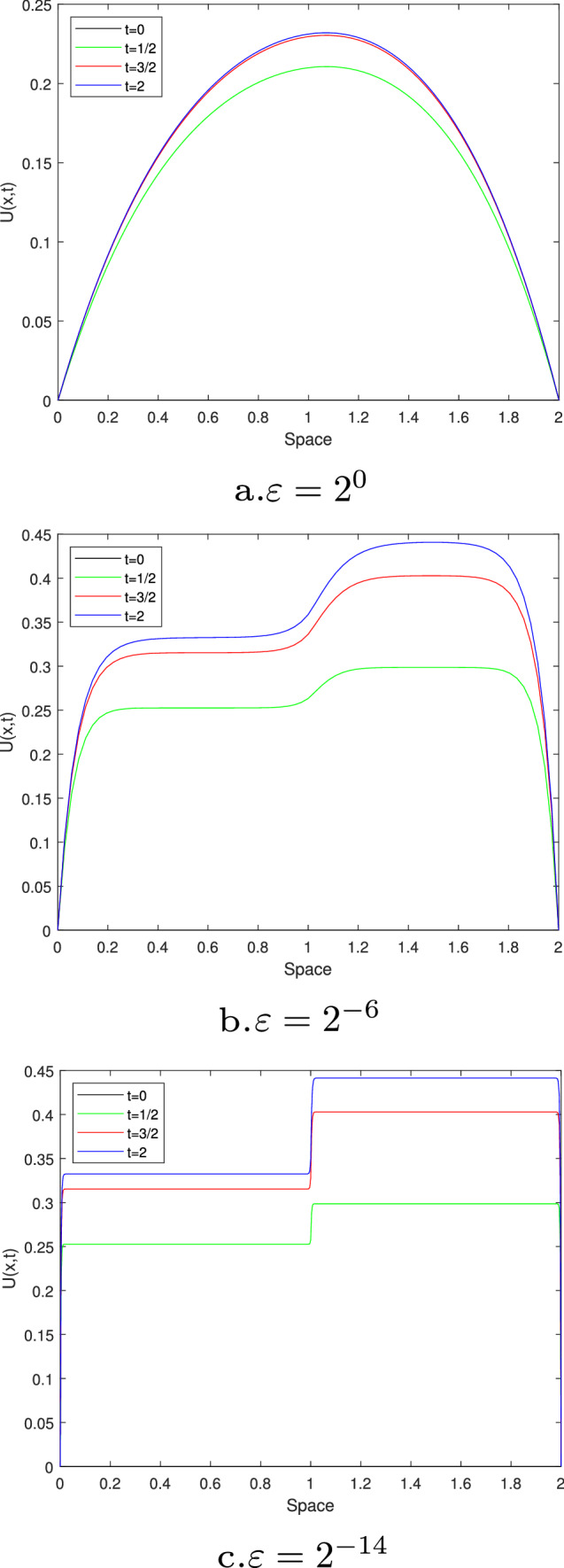
Simulations for the numerical solution of Example 1, taking $\theta$=0.5, $\beta$=1 and *N*=72 and *M*=72 at four different time levels (line plots)

**Fig. 2:**
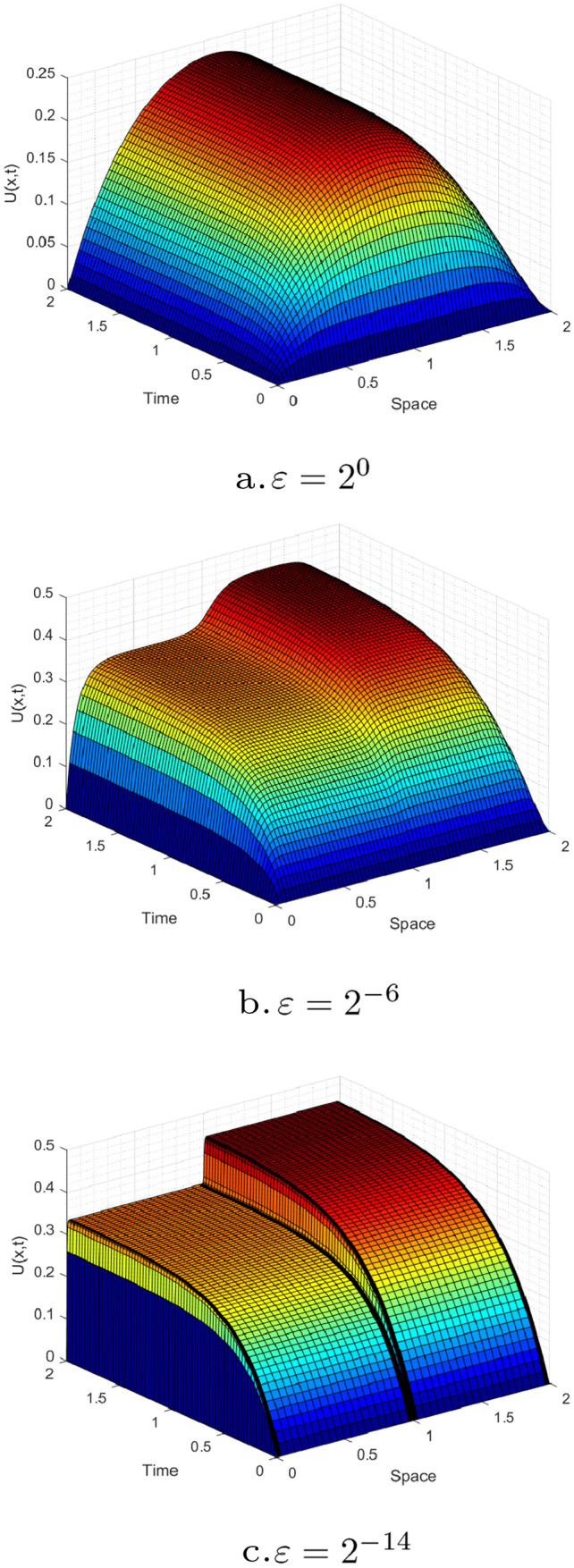
Simulations for the numerical solution of Example 2, taking $\theta$=0.5, $\beta$=1.5, *N*=64 and *M*=32 at different time levels and perturbation parameters (line plots)

**Fig. 3:**
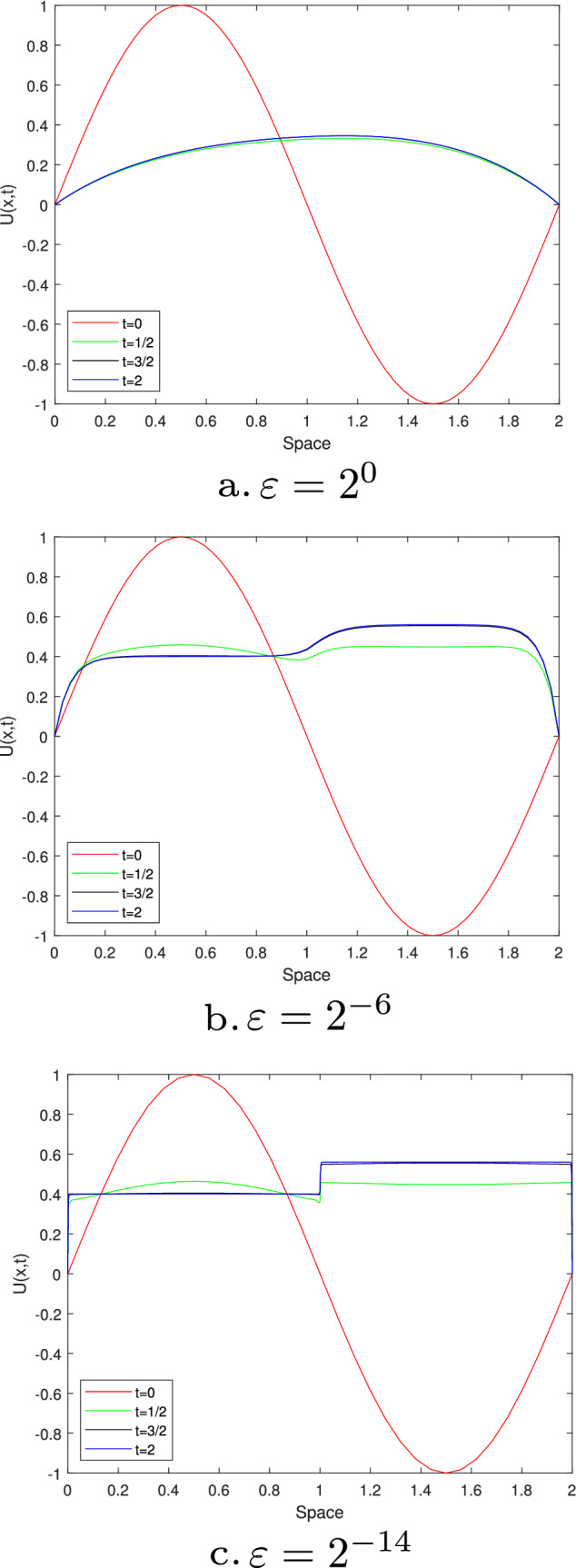
Simulations for the numerical solution of Example 1, taking $\theta$=0.5, $\beta$=1, *N*=72 and *M*=72 for different perturbation parameters (surface plots)

**Fig. 4:**
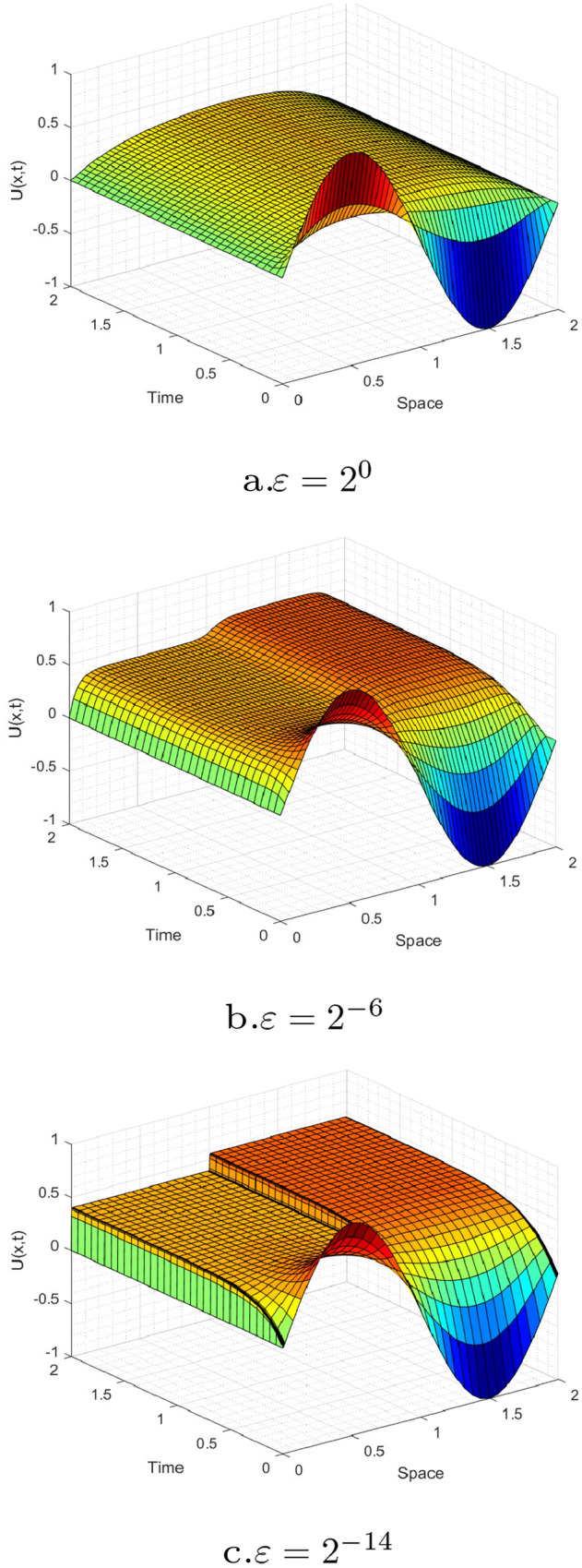
Simulations for the numerical solution of Example 2, taking $\theta$=0.5, $\beta$=1.5, *N*=64 and *M*=32 at different time levels and perturbation parameters (surface plots)

**Fig. 5:**
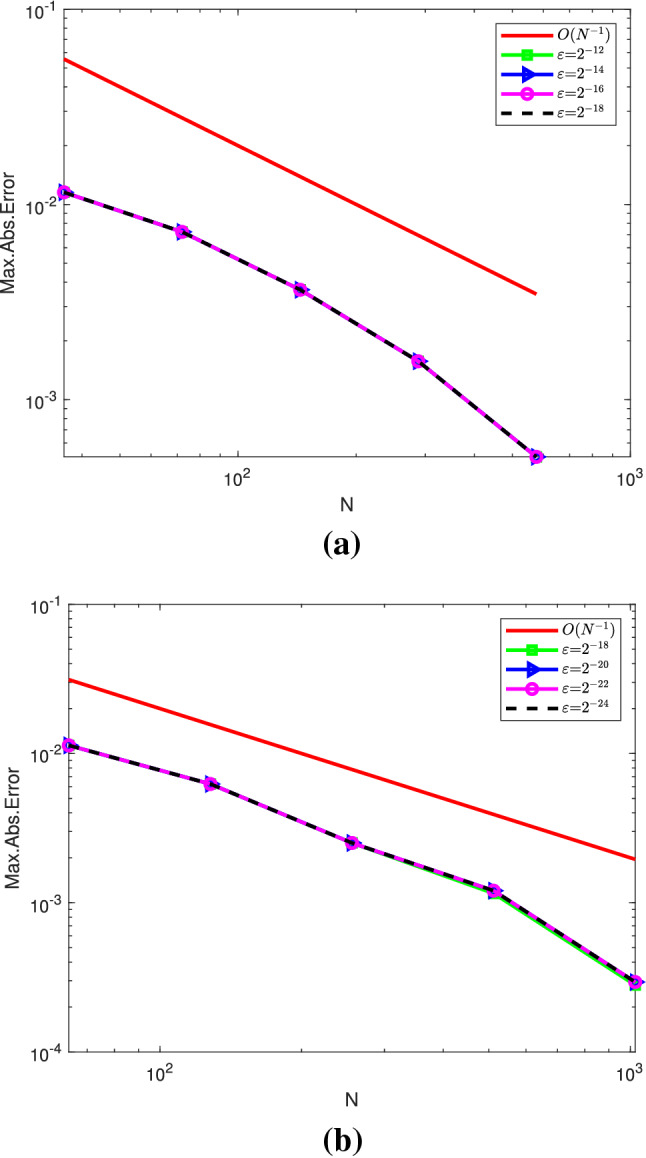
Log–log plots of maximum absolute error verses *N* with different values of $\varepsilon$ for (**a)** Example 1 and **(b)** Example 2

Figures 1 and 3 are plotted for Examples 1 and 2, respectively, at different time levels to show the changes in the boundary and interior layers with respect to the perturbation parameter. Also, to depict the physical behaviors of the solutions, surface plots are given in Figs. 2 and 4 for Examples 1 and 2, respectively. From these figures, we observe that as ε approaches to zero, the boundary and interior layers are resolved. To show the uniform convergence of the method, log-log plots are given in Fig. 5 for both examples.

**Table 1:** $E^{N,M}_{\varepsilon }$, $E^{N,M}$ and $R^{N,M}$ of Example 1 for $\beta = 1$ and $\theta =0.5$

$\varepsilon \,\,\,$	$N=36$	$N=72$	$N=144$	$N=288$	$N=576$
$\downarrow \,\,\,$	$M=36$	$M=72$	$M=144$	$M=288$	$M=576$
$2^{-00}$	3.4088e−03	1.6766e−03	8.4491e−04	4.2326e−04	2.1177e−04
$2^{-02}$	3.2042e−03	1.6160e−03	8.1425e−04	4.1563e−04	2.1031e−04
$2^{-04}$	1.4063e−03	1.1065e−03	7.5135e−04	4.0055e-04	2.0233e−04
$2^{-06}$	2.3392e−03	7.9088e−04	2.0311e−04	2.6921e−04	1.8661e−04
$2^{-08}$	5.9840e−03	2.7311e−03	7.9285e−04	2.0350e−04	5.1067e−05
$2^{-10}$	9.5374e−03	6.7288e−03	2.7339e−03	7.9334e−04	2.0359e−04
$2^{-12}$	9.5374e−03	7.2339e−03	3.6544e−03	1.5725e−03	5.0898e−04
$2^{-14}$	9.5374e−03	7.2339e−03	3.6544e−03	1.5725e−03	5.0898e−04
$E^{N,M}$	9.5374e−03	7.2339e−03	3.6544e−03	1.5725e−03	5.0898e−04
$R^{N,M}$	0.3988	0.9851	1.2166	1.6274

**Table 2:** $E^{N,M}_{\varepsilon }$, $E^{N,M}$ and $R^{N,M}$ for Example 2, taking $\beta =1.5$ and $\theta =0.5$

$\varepsilon \,\,\,$	$N=64$	$N=128$	$N=256$	$N=512$	$N=1024$
$\downarrow \,\,\,$	$M=32$	$M=64$	$M=128$	$M=256$	$M=512$
$2^{-00}$	2.9406e−02	6.9304e−03	2.0463e−03	9.6927e−04	4.7875e−04
$2^{-02}$	8.6368e−03	3.8818e−03	1.9143e−03	9.5221e−04	4.7773e−04
$2^{-04}$	6.8183e−03	3.6671e−03	1.8082e−03	9.4268e−04	4.7524e−04
$2^{-06}$	4.8601e−03	2.3395e−03	1.6507e−03	9.0401e−04	4.4682e−04
$2^{-08}$	6.6227e−03	2.1333e−03	5.7181e−04	5.5287e−04	4.0721e−04
$2^{-10}$	1.1337e−02	6.2471e−03	2.1336e−03	5.7187e−04	1.4558e−04
$2^{-12}$	1.1337e−02	6.2470e−03	2.5144e−03	8.5861e−04	2.7178e−04
$2^{-14}$	1.1336e−02	6.2469e−03	2.5144e−03	8.5860e−04	2.7178e−04
$2^{-16}$	1.1336e−02	6.2468e−03	2.5144e−03	1.0525e−03	2.7178e−04
$2^{-18}$	1.1336e−02	6.2468e−03	2.5144e−03	1.1547e−03	2.8047e−04
$2^{-20}$	1.1336e−02	6.2468e−03	2.5144e−03	1.2045e−03	2.9409e−04
$E^{N,M}$	2.9406e−02	6.9304e−03	2.5144e−03	1.2045e−03	4.7875e−04
$R^{N,M}$	2.0851	1.4627	1.0618	1.3311

**Table 3:** Comparisons of the present method with other results in literature

Example 1, when $\theta =0.5$ and $T=1$				
$N=M$:	36	72	144	288
Present method				
$E^{N,M}$	5.1103e−03	2.8812e−03	1.2004e−03	3.9571e−04
$R^{N,M}$	0.8267	1.2632	1.6010	
Results in [22]				
$E^{N,M}$	7.0100e−03	2.9700e−03	1.1400e−03	4.0600e−04
$R^{N,M}$	1.2390	1.3814	1.4895	

Example 2, when $\theta =0.5$ and $E^{2N,4M}$				
*N* :	64	128	256	512
*M*:	32	128	512	2048
Present method				
$R^{N,M}_{\varepsilon =2^{-20}}$	1.7710	1.8278	1.7093	1.6930
Results in [21]				
$R^{N,M}_{\varepsilon =2^{-20}}$	1.7908	1.8354	1.5091	1.6257

## Conclusion

In this research work, we considered a singularly perturbed differential equation with large delay in the spatial variable. Due to the influences of the perturbation parameter and the large delay, the solution of the problem changes rapidly in the layers, which is a challenging factor to solve the problem analytically. We treated the problem by constructing a numerical scheme. The scheme is obtained by approximating the time derivative term using $\theta$-method on a uniform mesh and the spatial derivative term is approximated using the central difference operator on a nonuniform Shishkin mesh. We established the stability and convergence analysis and obtained that the method is uniformly convergent. The method is demonstrated by solving two model examples. From results of the examples, we observe that the developed numerical scheme is convergent regardless of the perturbation parameter.

## References

[1] Humphries T (2016) Delay differential equations. University of Auckland, Bernd Krauskopf, NZMRI, pp 1–18

[2] Glizer V (2000). Asymptotic solution of a boundary-value problem for linear singularly-perturbed functional differential equations arising in optimal control theory. J Optim Theory Appl.

[3] Stein RB (1967). Some models of neuronal variability. Biophys J.

[4] Nelson PW, Perelson AS (2002). Mathematical analysis of delay differential equation models of HIV-1 infection. Math Biosci.

[5] Kiselev AIRKAF Ilya N (2021) A delay differential equation approach to model the COVID-19 pandemic. 10.1101/2021.09.01.21263002

[6] Rao RN, Chakravarthy PP (2014). A fitted Numerov method for singularly perturbed parabolic partial differential equation with a small negative shift arising in control theory. Numer Meth.

[7] Palli MMK, Ravi Kanth A (2021). Numerical simulation for a class of singularly perturbed convection delay problems. Khayyam J Math.

[8] Kanth AR, Kumar MM (2017). Numerical treatment for a singularly perturbed convection delayed dominated diffusion equation via tension splines. Int J Pure Appl Math.

[9] Narasimhan R (2016). Singularly perturbed delay differential equations and numerical methods. Singular perturbation problems. North-Holland Math Stud.

[10] Kumar D (2018). A collocation method for singularly perturbed differential-difference turning point problems exhibiting boundary/interior layers. J Differ Equ Appl.

[11] Kumar D, Kumari P (2020). A parameter-uniform collocation scheme for singularly perturbed delay problems with integral boundary condition. J Appl Math Comput.

[12] Cimen E, Amiraliyev GM (2019). Uniform convergence method for a delay differential problem with layer behaviour. Mediterr J Math.

[13] Cimen E (2020). Uniformly convergent numerical method for a singularly perturbed differential difference equation with mixed type. Bull Belgian Math Soc-Simon Stevin.

[14] Kumar P, Ravi Kanth A (2020). Computational study for a class of time-dependent singularly perturbed parabolic partial differential equation through tension spline. Comput Appl Math.

[15] Adilaxmi DB, Reddy YN (2019). An initial value technique using exponentially fitted Non standard finite difference method for singularly perturbed differential-difference equations. Appl Appl Math.

[16] Sahu Musharary JM S R (2020). A parameter uniform numerical scheme for singularly perturbed differential-difference equations with mixed shifts. J Appl Comput Mech.

[17] Shakti D, Mohapatra J (2021). Uniform convergence analysis of monotone hybrid scheme for convection-diffusion problems on layer adapted meshes. Math Rep.

[18] Bansal K, Sharma KK (2016). Parameter uniform numerical scheme for time dependent singularly perturbed convection-diffusion-reaction problems with general shift arguments. Numer Algorithms.

[19] Woldaregay MM, Duressa GF (2019). Parameter uniform numerical method for singularly perturbed parabolic differential difference equations. J Niger Math Soc.

[20] Ejere AH, Duressa GF, Woldaregay MM, Dinka TG (2022). An exponentially fitted numerical scheme via domain decomposition for solving singularly perturbed differential equations with large negative shift. J Math.

[21] Bansal K, Sharma KK (2018). Parameter-robust numerical scheme for time-dependent singularly perturbed reaction-diffusion problem with large delay. Numer Funct Anal Optim.

[22] Kumar D, Kumari P (2020). Parameter-uniform numerical treatment of singularly perturbed initial-boundary value problems with large delay. Appl Numer Math.

[23] Sahu SR, Mohapatra J (2021). Numerical investigation of time delay parabolic differential equation involving two small parameters. Eng Comput.

[24] Priyadarshana S, Mohapatra J, Govindrao L (2022). An efficient uniformly convergent numerical scheme for singularly perturbed semilinear parabolic problems with large delay in time. J Appl Math Comput.

[25] Govindarao L, Mohapatra J (2019). Numerical analysis and simulation of delay parabolic partial differential equation involving a small parameter. Eng Comput.

[26] Priyadarshana S, Mohapatra J, Pattanaik S (2022). Parameter uniform optimal order numerical approximations for time-delayed parabolic convection diffusion problems involving two small parameters. Comput Appl Math.

[27] Manikandan NMJVS M Shivaranjani (2014) A parameter-uniform numerical method for a boundary value problem for a singularly perturbed delay differential equation. In: Advances in applied mathematics, pp 71–88. 10.1007/978-3-319-06923-4-7

[28] Roos HG, Stynes M, Tobiska L (2008). Robust numerical methods for singularly perturbed differential equations: convection-diffusion-reaction and flow problems.

[29] Majumdar A, Natesan S (2019). An epsilon-uniform hybrid numerical scheme for a singularly perturbed degenerate parabolic convection-diffusion problem. Int J Comput Math.

[30] Kadalbajoo MK, Awasthi A (2011). The midpoint upwind finite difference scheme for time-dependent singularly perturbed convection-diffusion equations on non-uniform mesh. Int J Comput Methods Eng Sci Mech.

[31] Franklin MVSMJ V Paramasivam (2010) Second order parameter-uniform convergence for a finite difference method for a singularly perturbed linear parabolic system. http://arxiv.org/abs/1008.2470

[32] Woldaregay MM, Duressa GF (2021). Robust numerical method for singularly perturbed parabolic differential equations with negative shifts. Filomat.

[33] Clavero C, Gracia JL (2012). A high order HODIE finite difference scheme for 1D parabolic singularly perturbed reaction-diffusion problems. Appl Math Comput.

[34] Miller JJ, O’riordan E, Shishkin GI (2012). Fitted numerical methods for singular perturbation problems: error estimates in the maximum norm for linear problems in one and two dimensions.

[35] Doolan EP, Miller JJ, Schilders WH (1980). Uniform numerical methods for problems with initial and boundary layers.

